# When allergies have no name: is idiopathic anaphylaxis driven by co-factors?

**DOI:** 10.3389/falgy.2024.1468945

**Published:** 2024-10-18

**Authors:** Shuayb Elkhalifa, Haggar Elbashir, Mohamed Abuzakouk

**Affiliations:** ^1^ Allergy and Immunology Division, Cleveland Clinic Abu Dhabi, Abu Dhabi, United Arab Emirates; ^2^ Faculty of Biology, Medicine and Health, Centre for Musculoskeletal Research, The University of Manchester, Manchester, United Kingdom; ^3^ Department of Allergy and Clinical Immunology, Lancashire Teaching Hospitals NHS Foundation Trust, Preston, United Kingdom

**Keywords:** idiopathic anaphylaxis, mast cell tryptase, mast cell activation, mastocytosis, lipid transfer protein (LTP), hereditary alpha-tryptasemia, cofactors induced anaphylaxis, omega-5-gliadin

## Abstract

Idiopathic anaphylaxis (IA) is a severe allergic reaction without identifiable external triggers, presenting significant challenges in diagnosis and management. However, growing evidence suggests that many cases classified as idiopathic may actually be driven by cofactors such as exercise, hormonal fluctuations, medications, or hidden allergens. This mini-review explores the evolving understanding of IA, highlighting the role of these cofactors in triggering or amplifying anaphylactic reactions. It emphasizes how advances in diagnostic tools, including component-resolved diagnostics, are helping to identify previously undetected allergens, leading to more accurate diagnoses and reducing the prevalence of true idiopathic cases. As our knowledge of anaphylaxis and its underlying mechanisms deepens, the need for comprehensive evaluations that account for cofactor involvement becomes increasingly clear. Continued research in this area is essential to improve patient outcomes and better manage this complex condition.

## Definitions and epidemiology

Anaphylaxis is a systemic hypersensitivity reaction that is rapid in onset and can be fatal. It is typically triggered by specific allergens that cause mast cell and basophil activation, leading to the release of mediators like histamine, which result in the clinical manifestations of anaphylaxis (1, 2). However, in Idiopathic anaphylaxis (IA), no such triggers are identifiable despite extensive investigation (3).

Idiopathic anaphylaxis is a severe, life-threatening allergic reaction characterized by the sudden onset of symptoms without an identifiable external trigger (4, 5). Unlike typical anaphylaxis, which is triggered by known allergens such as foods, insect stings, or medications (1, 2), IA presents a significant diagnostic and management challenge due to its unpredictable nature. The pathogenesis of IA is not fully understood, making it a complex condition for both patients and healthcare providers (3).

The World Allergy Organization (WAO) defines anaphylaxis as a systemic hypersensitivity reaction characterized by rapid onset of life-threatening airway, breathing, or circulatory problems, often associated with skin and mucosal changes (6). IA is a subtype of anaphylaxis where no external trigger can be identified despite comprehensive evaluation (3, 7). The WAO specifies that IA is diagnosed when anaphylactic reactions occur without any identifiable external trigger, even after detailed patient history, thorough physical examination, and extensive diagnostic testing (6).

Epidemiologically, IA is considered rare but significantly impacts the quality of life of those affected. According to epidemiological studies in Europe (8) and United States (9) studies, the annual incidence of Anaphylaxis in the general population is approximately 0.03%–1.6%. Estimating the actual incidence and prevalence of IA is challenging due to variations in different reports (10–13). This variability likely arises from the use of different definitions or diagnostic criteria, diagnostic limitations in older studies (12, 13) (such as overlooked mast cell disorders), and potential over-diagnosis of IA by emergency departments. One indirect method to estimate IA incidence involves analyzing the percentage of anaphylaxis cases that remain idiopathic despite extensive evaluation by an allergist/immunologist (10, 11). Consequently, approximately 30%–60% of adult anaphylaxis cases and 10% of pediatric cases are deemed idiopathic after thorough evaluation (10–13). The demographic characteristics of patients with IA can vary, but studies (10–13) suggest a slight female predominance and a wide age range of onset (10–13). This broad demographic distribution further complicates the identification of potential underlying mechanisms and risk factors. Therefore, more extensive population studies are required to accurately determine the true prevalence, incidence, and demographics of idiopathic anaphylaxis (IA).

The psychological burden of anaphylaxis is substantial (14), with many patients reporting heightened anxiety, reduced quality of life, and the fear of experiencing severe episodes without warning in case of IA. These recurrent episodes can lead to significant healthcare utilization, including frequent emergency department visits and hospitalizations (14).

### Potential pathophysiological factors

The pathophysiology of idiopathic anaphylaxis (IA) is complex and remains poorly understood. Theories suggest that it involves an overactive immune response triggered by unknown internal or external factors. Several potential mechanisms have been proposed to explain the occurrence of IA (3):

### Mast cell disorders

Mast cells play a critical role in allergic reactions by releasing mediators such as histamine, prostaglandins, and leukotrienes upon activation (15). One of the compelling theory of IA pathogenesis proposes heightened mast cell activation in IA patients (11). This theory posits that IA patients possess hyperreactive mast cells that are more likely to degranulate due to the presence of extracellular Th2 cytokines (11). This hypothesis is supported by findings showing that IA patients have elevated levels of IL-4, IL-5, and IL-13 cytokines after lymphocyte stimulation compared to atopic individuals and healthy controls (16).

### Autoimmune processes

One possible theory for the pathogenesis of idiopathic anaphylaxis (IA) involves elevated numbers of activated lymphocytes (17). Studies (17, 18) have shown that IA patients exhibit a significantly higher percentage of activated T cells, characterized as CD3+ HLA-DR+, in their blood during acute episodes when compared to their baseline levels (17). This indicates a robust immune response during these episodes. Furthermore, these patients also demonstrate an increased number of activated B cells, identified as CD19+ CD23+ (17), not only during acute episodes but also at baseline, in comparison to control patients or those suffering from chronic idiopathic urticaria. This suggests a persistent state of heightened immune activation in IA patients.

Additionally, soluble Interleukin-2 receptor alpha chain (sCD25) levels, a marker of lymphocye activation, but also known to be cleaved in part from the mast cell surface are reported to be expressed by the bone marrow mast cells of patients with systemic mastocytosis but not healthy individuals, elevated plasma levels were noted in mastocytosis (median, 955 pg/mL; range, 138–2,829 in controls, vs. median, 2,491; range, 310–15,020 in mastocytosis; *P* < .00001) and in anaphylaxis (median, 1,361 pg/mL; *P* < .05 vs. controls) (18). Further studies are required to understand the use of sCD25 in the context of IA.

### Hormonal cofactors in idiopathic anaphylaxis

Recent studies have highlighted the role of hormonal cofactors in the development of anaphylaxis, particularly the effects of sex hormones. These findings suggest that hormonal fluctuations, especially during menstruation or pregnancy, can increase the risk of anaphylaxis. The review by Untersmayr et al. (19) discusses sex hormone-related hypersensitivity and clinical approaches for managing these reactions, while Salvati et al. (20) and other studies provide further evidence of gender differences in anaphylaxis. This underscores the importance of considering hormonal influences in diagnosing and managing idiopathic anaphylaxis. The role of hormonal cofactors has expanded our understanding of anaphylaxis triggers, moving beyond traditional allergens to include physiological and biochemical conditions that may predispose patients to severe reactions.

### Genetic factors

Hereditary alpha-tryptasemia (HαT) is an autosomal dominant inherited genomic variant characterized by the duplication or multiple copy numbers of the α-tryptase gene (TPSAB1), resulting in an increased number of mast cells (MCs) in bone marrow biopsy specimens (21). Despite this increase in mast cell numbers, HαT generally does not lead to elevated urinary secretion of other mast cell mediators. This genetic variant is relatively common, found in approximately 6% of the general population, with serum baseline tryptase (sBT) levels typically exceeding 8.0 ng/mL (21).

Patients with HαT may exhibit a diverse clinical phenotype, including symptoms reminiscent of Ehlers-Danlos syndrome, such as joint hypermobility with arthritis, postural orthostatic tachycardia syndrome (POTS), flushing, gastrointestinal hypomotility, vibratory urticaria, irritable bowel syndrome, eosinophilic esophagitis, neuropsychiatric diagnoses, chronic musculoskeletal pain, and allergic disorders affecting the cutaneous, respiratory, or cardiovascular systems (3, 21). However, it is important to note that these symptoms might be subject to referral bias, and there has been inconsistency in the clinical phenotype observed in unselected cohorts. The etiology of the symptom complex associated with HαT remains unknown.

Research indicates that patients with HαT have an increased risk of severe spontaneous and insect venom-triggered anaphylaxis episodes (3, 21). Notably, a recent study (22) found that increased germline copies of α-tryptase are associated with a higher severity of venom anaphylaxis. These increased copy numbers are more prevalent among individuals with idiopathic anaphylaxis and systemic mastocytosis (SM) and are linked to a heightened relative risk of anaphylaxis among patients with SM (22). Therefore, HαT may independently confer an increased risk for severe anaphylaxis, separate from any concomitant clonal mast cell disorders.

However, these findings need to be confirmed in larger patient cohorts, as to date, no studies have conclusively shown that mast cells in patients with HαT are hyperresponsive. This ongoing research is crucial to fully understand the implications of HαT in the context of mast cell and IA and to develop targeted interventions for managing this complex condition.

## Cofactors and anaphyalxis

Our hypothesis is that cofactors may play a central role in the majority of idiopathic anaphylaxis (IA) cases, particularly those where identifiable triggers have not yet been determined. Emerging evidence suggests (23–25) that factors such as physical stress, medications, or latent allergens may act as primary contributors in precipitating anaphylactic events. However, while this theory presents a compelling framework for understanding IA, it remains speculative at this stage. Comprehensive studies and rigorous clinical trials are necessary to further investigate and validate this concept, as well as to determine the specific mechanisms by which cofactors influence anaphylactic reactions in the absence of apparent triggers.

### Exercise and anaphylaxis

Exercise-induced reactions (urticaria &/or anaphylaxis) are relatively rare conditions; patient with this disorder develop an IgE mediated allergic reactions in conjunction with cofactors or physical activities that subsequently may result in anaphylaxis. The lifetime prevalence of exercise-induced reactions is thought to be about 0.05%; and around 30%–50% of these patients may be food dependent (24, 26–29) i.e., only develop the reaction in the context of food and exercise combined.

### Food and anaphylaxis

The primary allergen in wheat-dependent exercise-induced anaphylaxis (WDEIA) is ω5-gliadin, a component of the gluten fraction in wheat (25). Testing for ω5-gliadin-specific IgE (sIgE) using the ImmunoCAP® assay (Phadia, Uppsala, Sweden) has shown high sensitivity (up to 80%) and specificity (approximately 96%) (25, 30). Consequently, the term “ω5-gliadin allergy” is often used synonymously with WDEIA. However, other allergens are also relevant in this disease.

In addition to ω5-gliadins, high-molecular-weight glutenin subunits (28) (HMW-GS) are significant allergens in WDEIA. Other potential allergens from the wheat gluten fraction include low-molecular-weight glutenin subunits (LMW-GS) and α/β/γ-gliadins (25, 30–32).

Among non-gluten allergens, some cases of ω5-gliadin-negative WDEIA may be caused by wheat lipid transfer protein Tri a 14 (25, 30–32). Unlike ω5-gliadins, asymptomatic sensitization to Tri a 14 is common, making oral challenge tests indispensable for diagnosing Tri a 14-WDEIA. Other features of Tri a 14-WDEIA include a stronger association with atopy and higher cross-reactivity with other foods such as nuts or other cereals.

Another subtype of ω5-gliadin-negative WDEIA is due to percutaneous sensitization to hydrolyzed wheat proteins (HWP), often used in soaps, shampoos, and other cosmetics. In Japan, over 1,300 patients developed HWP-WDEIA after using HWP-containing soap. Initially, these patients experienced local symptoms, which later progressed to WDEIA symptoms, most commonly eyelid edema, but also anaphylaxis after ingestion of natural wheat products combined with cofactors (30–32). Similar cases have been reported in Europe due to cosmetic use (25, 30–32).

More recently, a new subtype of ω5-gliadin-negative WDEIA (31) related to grass pollen allergy has been proposed. Although asymptomatic cross-sensitization to wheat is common (approximately 65%) in grass pollen-allergic subjects, Ogino and colleagues reported that strong grass pollen sensitization could cause ω5-gliadin-negative WDEIA in individual patients (not caused by HWP or Tri a 14), possibly due to cross-reactivity of peroxidase-1 and beta-glucosidase between grass pollen and wheat.

In a Japanese study (33), the prevalence of wheat allergy in adults was found to be 0.21%. Other cofactors such as alcohol, nonsteroidal anti-inflammatory drugs (NSAIDs), infection, stress, and female sex hormones/menstruation (34) can also act as a cofactor to induce a reaction.

The first published case of a patient who developed anaphylaxis during exertion was after ingestion of shellfish (35), the condition was termed Food dependent exercise induced anaphylaxis (FDEIA). A number of food items have been implicated in FDEIA such as wheat, soya, peanut, milk and sea food (36). Anaphylaxis might also occur following exercise without food intake reported originally by Sheffer and Austen in 1980 (26) and this is called exercise induced anaphylaxis (EIA). EIA constitutes only a 5%–15% of all cases of anaphylaxis (28), whilst a third to a half of EIA are due to FDEIA (28).

In a patient with suspected WDEIA, many variables, such as the amount of allergen ingested, type of physical activity, environmental factors, concomitant medications/drugs, emotional stress and fatigue, can affect the nature of events that occur (25). The type of physical activity required for eliciting a reaction is variable and the level of exercise can be minimal, for example walking to get a bus.

### Pathophysiology of WDEIA/FDEIA

The pathophysiological pathways of WDEIA/FDEIA are not fully understood; there are more to uncover, which will require further research. In broad terms, there have been few theories to explain these mechanisms, which are summarised below (25, 37):

- Alterations in plasma osmolality;
- Alterations in plasma pH;
- Alterations in tissue enzyme activity;
- Alterations in blood flow redistribution;
- Alterations in gastrointestinal permeability;
- Facilitated epitope recognition/allergen binding.

#### Alterations in plasma osmolality

Alterations in osmolality can increase the activation of basophils; hence they have suggested the same mechanisms might occur in patients with WDEIA/FDEIA (26, 29). Studies on basophils of patients with FDEIA compared to healthy controls have shown increased histamine release in the FDEIA patient at higher osmolality compared to control groups (29).

#### Alterations in plasma pH

Mast cell enzymatic homeostasis is entirely dependent on an acidic environment (38). Various studies have shown that mast cell activation is directly affected by alterations in serum pH (38, 39). Katsunuma et al. (38) have reported a case of WDEIA where they have used sodium bicarbonate to inhibit anaphylactic symptoms following wheat and exercise provocation, they have shown the reduction in serum pH in comparison to increase in histamine levels was also inhibited.

#### Alterations in tissue enzyme activity

Palosuo et al (40) have shown O5G–derived peptides are cross-linked by (tissue-transglutaminase) tTG, which also causes an increase in IgE binding capacity. In addition activation of tTG during exertion in the gastrointestinal mucosa of various cases with WDEIA may lead to the formation of large complexes which might be capable of eliciting anaphylactic reactions.

Another theory that an increase in IL 6 during exercise may upregulate tTG, which in turn can cause aggregation of O5G, the new compound might be more capable of efficiently cross link Fcε receptors on basophils and mast cells (40).

#### Alterations in blood flow redistribution

Exercise increases blood flow to skeletal muscle and skin, whilst decreasing the gastrointestinal mucosal blood flow. This may increase the concentration of allergens at skin and musculoskeletal sites; which then results in exposing mast cells at these sites to higher levels of allergens (25, 28–30).

#### Alterations in gastrointestinal permeability

The tight junctions in the gut epithelium have the ability to become permeable on exposure to various stimuli and injuries, which may include alcohol ingestion and NSAIDs. This may result in leakage at the intestinal barrier, which might increase the risk of developing anaphylaxis (25, 41). The effect of exercise on gut permeability may also result in adverse reactions, this have been reported in athletes exposed to high-intensity exercise (18, 21). The intensity and duration of exercise might play a role, in addition to the effect of blood flow redistribution.

#### Facilitated epitope recognition/allergen binding

Once IgE cross-links with a specific food allergen and, when combined with physical activities and exercise, the threshold for mast cell and basophil degranulation may reduce, hence the release of histamine and vasoactive mediators may in turn lead to anaphylaxis (25, 30, 42). It has been also shown also by Wolbing et al. (42) that exercise reduces the threshold for mast cell and basophil activation.

The diagnosis of WDEIA is not straightforward, especially with regards to the reproducibility of symptoms.

### Do familial phenotypes exist?

There is a growing body of evidence suggesting that epidermal sensitization due to epidermal barrier defects play a significant role in systemic allergic reactions such as WDEIA (43, 44). Filaggrin (FLG) is an integral epidermal protein in skin barrier function and there are multiple studies confirming that mutations in the gene encoding filaggrin predispose to the development of atopic eczema and other allergic disorders such as asthma, allergic rhinitis and IgE mediated food allergies (45). These studies may strengthen the theory that skin barrier dysfunction predispose to epidermal sensitization and hence the development of allergies such as WDEIA. Sandilands et al. (46) & others have described that∼45% of young patients with atopic dermatitis and carry FLG mutations exhibit a higher prevalence of food allergies. Sampson (47) has shown that IgE-mediated peanut allergy is strongly associated with FLG mutations, with an odds ratio of 5.3 (*P* = 3.0 × 10−6; 95% CI, 2.8–10.2) (47). A case report of a Japanese family (45) with two individuals carrying FLG null mutations developed WDEIA. This is the first detailed report of a possible familial case of WDEIA. However more studies are required to investigate this theory further to elaborate the pathogenesis of this condition.

#### Alpha-gal syndrome

Recent studies have indicated a rising incidence of mammalian red meat allergy and anaphylaxis linked to an exposure to the mammalian oligosaccharide, galactose-α-1,3-galactose (α-gal) (48). Consequently, numerous individuals previously categorized under idiopathic anaphylaxis (IA) have been reclassified as having α-gal syndrome. Distinct from typical IgE-mediated food reactions, allergy to mammalian meat can exhibit symptoms delayed by up to six hours post-consumption (48). This lag is believed to stem from the time required for digestion to reveal the carbohydrate epitope (3). Additionally, the responsiveness to α-gal can be heightened by factors like exercise, alcohol, or aspirin (49). This delay in symptom onset had previously obscured the true cause of anaphylaxis, leading to misdiagnosis as IA before α-gal was identified (3, 48, 49). Notably, a study involving 70 patients originally diagnosed with IA found that upon further investigation, six patients, including two with mastocytosis, exhibited reactions linked to α-gal sensitization (50). It is important to consider that not all individuals sensitized to α-gal will exhibit clinical symptoms; therefore, the clinical relevance of detecting α-gal-specific IgE (α-gal-sIgE) remains somewhat ambiguous. Further research is necessary to determine how α-gal-sIgE levels correlate with actual allergic reactions and to understand the extent to which this biomarker can reliably predict clinical outcomes in sensitized patients. This ambiguity underscores the need for a clearer diagnostic framework to assess the true significance of α-gal-sIgE in the context of diagnosing and managing α-gal syndrome.

### Diagnostic approach

**Figure 1:**
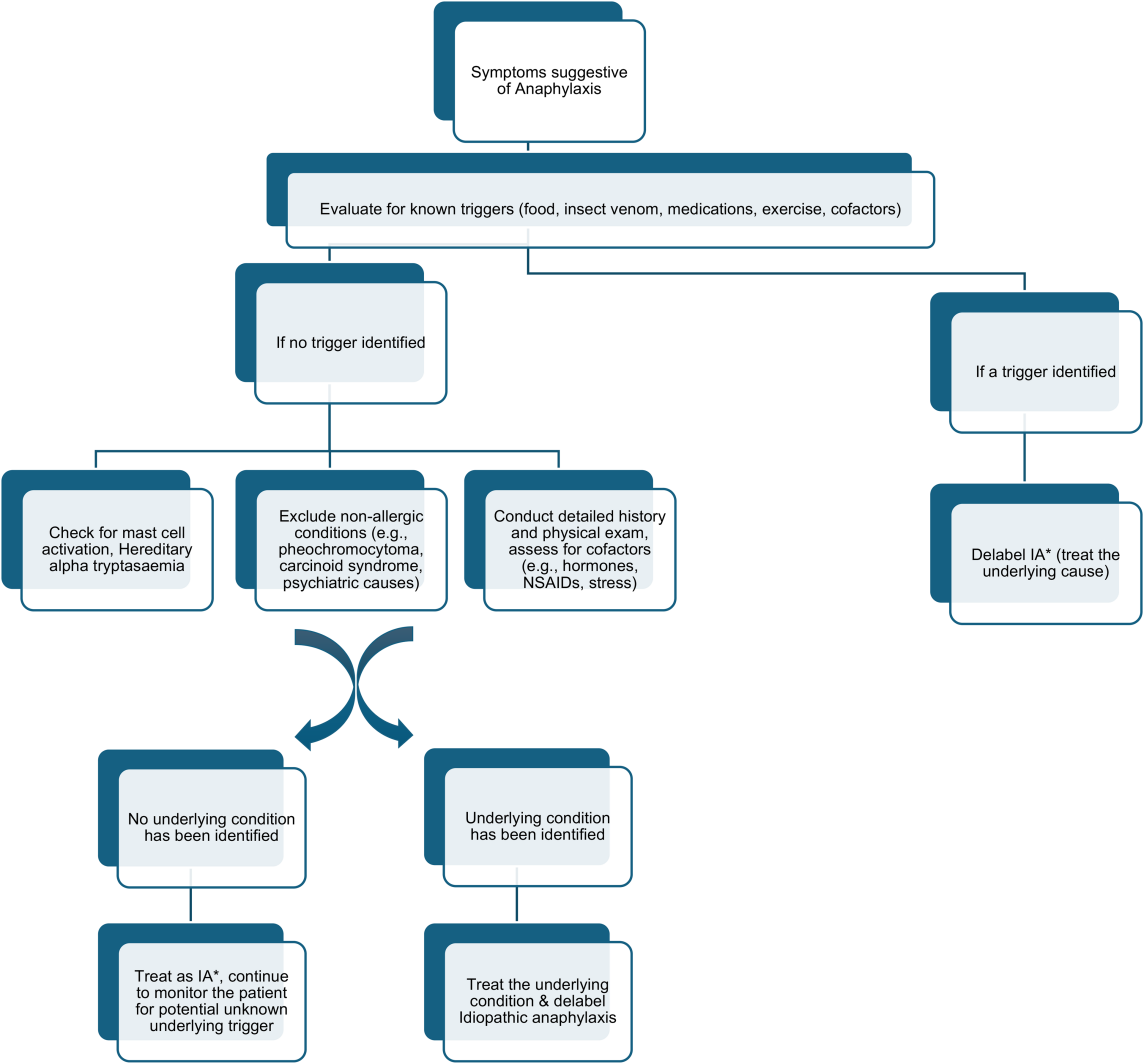
A proposed diagnostic pathway for idiopathic anaphylaxis (IA*).

The differential diagnosis of IA is extensive and encompasses a wide range of potential triggers and conditions that may present with similar symptoms (7, 23). Below flow chart (Figure 1) illustrate a proposed diagnostic pathway for IA. To start with, all known causes of anaphylaxis must be considered, including reactions to hidden and novel allergens in food, hymenoptera venom (such as bee or wasp stings), medications, and physical exertion or other cofactors. Each of these can precipitate anaphylactic responses that may initially appear idiopathic until a specific trigger is identified.

Moreover, other allergic conditions should be included in the differential diagnosis. Acute urticaria and angioedema, which involve localized skin reactions and swelling, can sometimes mimic the systemic nature of anaphylaxis. Similarly, acute asthma episodes, primarily affecting the respiratory system, can present with breathing difficulties that resemble those seen in anaphylaxis (23).

In addition to allergic reactions, several endocrine disorders must be ruled out. Conditions such as pheochromocytoma and carcinoid syndrome can cause flushing and other symptoms that overlap with anaphylactic reactions. These disorders involve the overproduction of certain hormones, leading to episodic symptoms that can be confused with anaphylaxis.

Cardiovascular conditions also play a role in the differential diagnosis. Postural orthostatic tachycardia syndrome (POTS) can cause symptoms like rapid heartbeat and dizziness upon standing, which may be mistaken for an anaphylactic reaction. It is crucial to differentiate between these conditions to provide appropriate treatment (3, 23).

Psychiatric conditions should not be overlooked, as they can present with physical symptoms that mimic anaphylaxis. Panic disorders, for instance, can lead to sudden episodes of intense fear accompanied by physical symptoms such as shortness of breath, palpitations, and dizziness. Somatoform disorders, where psychological distress manifests as physical symptoms, should also be considered (3, 7, 23).

Therefore, when diagnosing IA, it is imperative to conduct a thorough evaluation to rule out these other potential causes. This comprehensive approach ensures that the true underlying condition is identified, leading to more effective and targeted management of the patient's symptoms.

## Conclusion

While idiopathic anaphylaxis (IA) is defined by the absence of identifiable external triggers, emerging research strongly suggests that many cases previously classified as idiopathic are, in fact, driven by cofactors. These cofactors—ranging from exercise and hormonal fluctuations to medications and hidden allergens—play a pivotal role in triggering or amplifying anaphylactic responses.

As diagnostic tools such as component-resolved diagnostics advance and our understanding of cofactor involvement deepens, the prevalence of true idiopathic anaphylaxis is expected to shrink. The identification of novel allergens, like those responsible for alpha-gal syndrome and LTP hypersensitivity, along with greater recognition of the importance of cofactors like exercise and hormonal changes, has already begun to reclassify many cases initially thought to be idiopathic.

Ultimately, this evolving knowledge underscores the need for comprehensive evaluations that include a detailed assessment of potential cofactors. With continued research and improved diagnostic precision, clinicians will be better equipped to manage these complex cases, reducing the burden of uncertainty for patients and enhancing treatment outcomes.
